# Use of Digital and Telemedicine Tools for Postoperative Pain Management at Home: A Scoping Review of Health Professionals’ Roles and Clinical Outcomes

**DOI:** 10.3390/jcm14114009

**Published:** 2025-06-05

**Authors:** Gianluca Azzellino, Ernesto Aitella, Lia Ginaldi, Patrizia Vagnarelli, Massimo De Martinis

**Affiliations:** 1 Department of Life, Health and Environmental Sciences, University of L’Aquila, 67100 L’Aquila, Italy; ernesto.aitella@graduate.univaq.it (E.A.); lia.ginaldi@univaq.it (L.G.); demartinis@cc.univaq.it (M.D.M.); 2 Complex Operational Unit, Adriatic District Area, Azienda Unità Sanitaria Locale 04 Teramo (AUSL 04 Teramo), 64100 Teramo, Italy; patrizia.vagnarelli@aslteramo.it; 3 Allergy and Clinical Immunology Unit, Center for the Diagnosis and Treatment of Osteoporosis, Azienda Unità Sanitaria Locale 04 Teramo (AUSL 04 Teramo), 64100 Teramo, Italy; 4 Long-Term Care Unit, “Maria SS. dello Splendore” Hospital, Azienda Unità Sanitaria Locale 04 Teramo (AUSL 04 Teramo), Giulianova, 64021 Teramo, Italy; 5 UniCamillus-Saint Camillus International University of Health Sciences, 00131 Rome, Italy

**Keywords:** postoperative pain, telemedicine, digital health, pain management, healthcare professionals, home care

## Abstract

Postoperative pain management after hospital discharge remains one of the main clinical challenges. The use of digital and telemedicine tools offers new opportunities for the continuous monitoring of, and timely intervention in, patients discharged and followed at home. This scoping review, conducted according to the PRISMA-ScR checklist and the Joanna Briggs Institute methodology, analyzed 26 studies selected through a search of PubMed, Scopus, and Web of Science databases. Inclusion criteria comprised studies published between 2015 and 2025 that involved patients discharged home after surgery, that used digital or telemedicine tools for pain management, and that included active involvement of healthcare professionals and reported clinical outcomes. Studies show the use of a variety of digital tools, including mobile applications, web platforms, wearable sensors, automated messaging systems, and virtual reality technologies, alternating across settings for the assessment and management of pain at home, educational and therapeutic support, and to enhance communication between healthcare professionals and patients. Most reported outcomes focus on improved home-based pain control, a reduction in opioid consumption, and a high level of patient satisfaction. However, some challenges remain, particularly the low level of digital literacy among certain segments of the population. In conclusion, the implementation of telemedicine and digital technologies for managing postoperative pain at home proves to be a promising strategy. Nonetheless, it requires further scientific investigation and, from policymakers, significant investments in professional training and technological infrastructure to ensure an increasingly equitable and sustainable distribution of home healthcare services.

## 1. Introduction

Pain management at home is one of the main causes of discomfort and complications in the postoperative period, with a significant impact on patient well-being, the risk of hospital readmission, and the quality of post-surgical recovery. The increasingly shorter hospital stays and the changes in organizational needs following the COVID-19 pandemic have shifted the responsibility for pain management from the hospital setting to the home environment, making the activation of advanced remote support technologies essential [1,2,3]. In this context, advanced digital tools and telemedicine are offering important opportunities with the aim of improving care continuity, effective hospital-to-home transition, active symptom monitoring, and support for therapeutic adherence through fast and real-time communication [4,5]. Mobile apps, non-invasive sensors, web platforms, and integrated messaging systems are just some examples of tools that have proven effective in reducing pain at home, while also improving perceived clinical outcomes and preventing complications related to poor postoperative pain management at home [4,6]. Digital tools have enabled direct involvement of both patients and caregivers, enhancing self-management and the sense of safety during home [1,3]. Digital applications, such as those described by Debono et al. [5] and Gille et al. [7], have significantly reduced the need for unscheduled visits while ensuring high patient satisfaction, optimizing healthcare resources, and reducing waste. However, the effectiveness of these tools is closely linked to the role and capabilities of healthcare professionals, who must be able to integrate clinical-care pathways with new digital technologies [8,9]. The new global healthcare organization calls for a rethinking of care models, which includes highly specific training for staff and the enhancement of advanced competencies [7,10]. With the rise of outpatient surgery and “same-day discharge” [2], the need for structured follow-up pathways based on digital systems is growing. All these factors present organizational and professional challenges for nursing and healthcare staff, who are at the center of this transformation. The integration of new technologies [10], the reorganisation of home care services, the evolving role of professional leadership [11], and the impact of missed nursing care on job satisfaction and intention to leave the profession [12] are shaping the path toward the implementation of new care paradigms that must be carefully and systematically studied. Despite the extensive literature available, there are still critical issues in the management of pain and quality of life during the early post-discharge period, which are often overlooked in follow-up assessments [8]. There is limited evidence on the impact and effectiveness of assisted same-day discharge programs on pain and opioid use [13], and a limited clinical application of predictive and personalized models to guide targeted digital interventions, especially in pediatric settings [14].

The aim of this scoping review is to systematically map the existing literature on the use of digital and telemedicine tools for the management of postoperative pain at home.

## 2. Materials and Methods

This scoping review was conducted following the PRISMA-ScR checklist [13] and the methodology proposed by the Joanna Briggs Institute (JBI) for scoping reviews [14]. In addition, a recent methodological guidance proposed by Peters et al. [15] was followed, with the aim of ensuring a rigorous, systematic, and transparent synthesis. The review was not registered, as registration is not a mandatory requirement for this type of study. However, all methodological steps were carried out rigorously, including the definition of eligibility criteria, the search strategy, data extraction, and data synthesis. The review was guided by the following research questions:

- What digital or telemedicine tools are used for managing postoperative pain at home?
- What is the role of healthcare professionals?
- What clinical outcomes result from the use of these interventions?

To guide the definition of inclusion criteria, the PCC framework (Population, Concept, Context) was applied:

Population: patients (children, adults, or older adults) who have undergone surgical procedures and are followed up at home after hospital discharge.

Concept: use of digital health tools and telemedicine for the assessment and management of postoperative pain.

Context: post-discharge home care, including follow-up interventions through telemedicine, mobile applications, wearable devices, or other digital systems.

### 2.1. Databases Used and Search Strategy

For the development of the scoping review, the following databases were consulted: PubMed, Scopus, and Web of Science (Supplementary File S1). The literature search was conducted up to 30 May 2025. No additional search strategies were employed.

### 2.2. Data Extraction

Referring to the JBI methodology for scoping reviews [16], a results extraction table was created for the included articles. The table and its data extraction were developed using a standardized form that included the following items: author/year, main theme, geographical context, study design and methods, population and sample characteristics, key findings, and research gaps. To ensure consistency, sample size was considered a research gap when the study included fewer than 50 participants or was explicitly identified as a pilot or feasibility study by the authors.

### 2.3. Study Selection

Included studies met the following criteria:

- Addressed the use of digital or telemedicine tools for managing pain at home after surgical procedures;
- Involved healthcare professionals in home monitoring and intervention processes;
- Reported clinical outcomes resulting from the use of such tools, including data related to pain or treatment adherence;
- Were published in English and dated between 2015 and 2025.

Studies were excluded if they:

- Did not focus on postoperative pain in patients;
- Did not involve home monitoring;
- Did not describe digital tools or telemedicine used in the interventions.

### 2.4. Screening Process

To ensure the accurate inclusion of studies relevant to the review, the selection process was divided into two phases.

**Figure 1:**
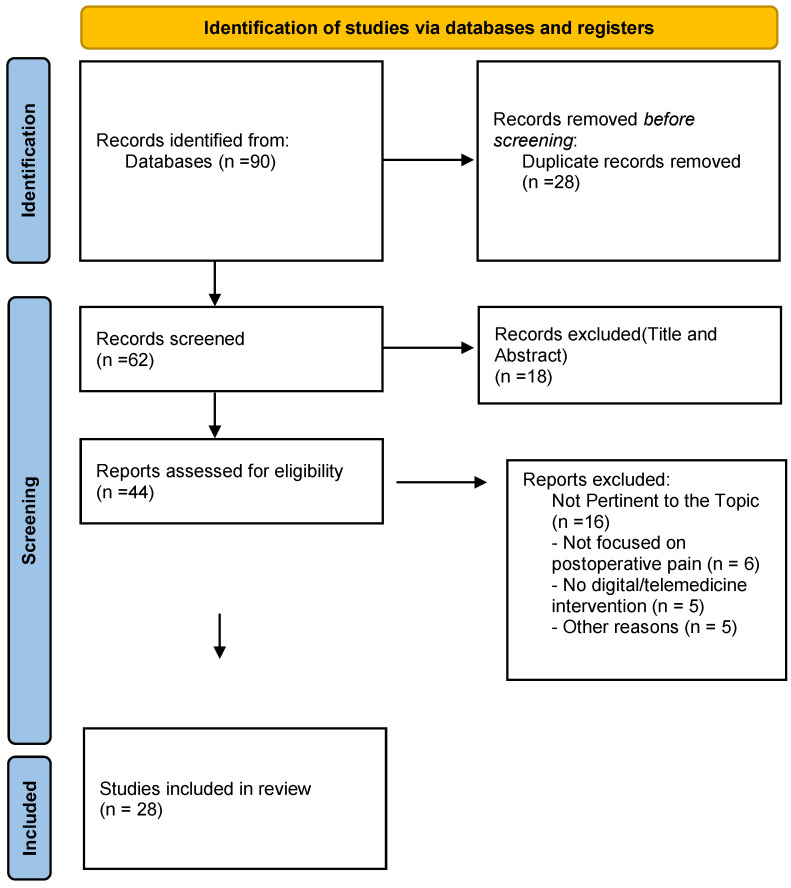
PRISMA 2020 flow diagram illustrating the study selection process. The databases searched were PubMed, Scopus, and Web of Science.

In the first phase, two independent reviewers screened the titles and abstracts of the articles identified through the search strategy, based on the predefined inclusion and exclusion criteria. In the second phase, the full texts of the selected studies were read to confirm their eligibility for inclusion. All studies were managed using Zotero (https://www.zotero.org/, last accessed 7 April 2025), which assisted the authors in organizing citations and full texts throughout the scoping review process. Discrepancies between the two reviewers were resolved with the involvement of two additional reviewers. The selection process is illustrated in the PRISMA 2020 flow diagram (Figure 1), in accordance with the PRISMA-ScR protocol [13].

### 2.5. Quality Assessment and Risk of Bias

The risk of bias assessment was not performed for this study, as it is not required for a scoping review. The main objective of this study was to analyze and map the available evidence, rather than to critically evaluate each individual study. The authors’ decision is consistent with the methodological guidelines for scoping reviews, as outlined in the JBI Manual for Evidence Synthesis and the PRISMA-ScR.

### 2.6. Data Synthesis

The synthesis of the results from the included studies follows the approach out-lined in the JBI Manual for Evidence Synthesis (2020) for scoping reviews. Through a descriptive synthesis, without critical appraisal of methodological quality, the data were extracted and organized, as recommended for this type of review. The synthesis in this study was structured into three main phases. In the first phase, following data extraction, two independent reviewers identified the key themes. They then analyzed the content of the included studies to identify the digital and telemedicine tools used, the role of healthcare professionals, and the main outcomes related to postoperative pain management at home.

In the second phase, the data were grouped thematically. The main emerging areas included: the type of technology used (e.g., mobile apps, sensors, video calls, automated messaging), the modes of involvement of healthcare personnel, and clinical outcomes such as reported pain, readmissions, and patient satisfaction.

In the third phase, aiming to maintain fidelity to the original data, the findings were qualitatively described and synthesized. The authors provided a comprehensive overview of the available evidence on the topic, highlighting both the limitations and potential of technologies used in the home-based management of postoperative pain.

To support and refine the language and style of the manuscript, the authors used Artificial Intelligence (AI), specifically ChatGPT-4o (OpenAI, San Francisco, CA, USA). All methodological steps and scientific decisions were made solely and exclusively by the authors.

## 3. Results

### 3.1. Selection of Studies

The initial search of electronic databases yielded a total of 90 studies, of which 28 were removed as duplicates. A total of 62 studies were screened, and after reviewing titles and abstracts, 18 studies were excluded, resulting in 44 articles selected for full-text assessment. Of these, 16 studies were excluded because they were not relevant to the objective of the scoping review. At the end of the selection process, 28 studies were included in the final analysis (Table 1).

### 3.2. Main Results

The following results derive from a narrative and qualitative synthesis of the included studies, based on the descriptive extraction of their key findings.

From the review of the 28 included studies, a diversity emerged both in terms of the technological tools used and the clinical settings, but all shared the common goal of monitoring and managing postoperative pain at home through telemedicine interventions or the use of various digital tools.

- Digital Tools Used

Almost all of the studies used mobile smartphone applications as the main tool for home follow-up [17,32]. Some studies integrated were web-based [6,8], while others employed systems based on automated or robotic messaging [7,16]. Some studies incorporated mobile platforms for collecting Patient-Reported Outcomes (PROs) [18]. These scenarios allow for the personalization of pain management at home following surgical procedures [14]. Advanced and sophisticated technologies also emerged in several studies, such as wireless sensors for passive monitoring of physiological parameters [22], or systems based on virtual and augmented reality [30], which showed promising results in reducing pain through multisensory distraction techniques. Other studies focused on the effectiveness of regularly scheduled follow-up phone calls [13]. In orthopedic care settings, some studies reported the use of tele-visits as a replacement for in-person outpatient visits [21], with similar and comparable results in terms of pain management and monitoring, and with high satisfaction and preference expressed by patients for the remote visit system. Excellent results were observed in pediatric settings, where simple and intuitive mobile applications proved highly effective for home pain monitoring, also enabling active involvement of caregivers, represented in these cases by the parents [25].

- Role of Healthcare Professionals

The studies show strong involvement of healthcare professionals: from active monitoring with alerts in case of severe symptoms and timely contact, to the evaluation and response to patient feedback, and even participation in the development and implementation of the technology itself [14]. In some settings, patients could directly initiate contact with a clinician through an app, which proved particularly useful for pain management [1]. In others, the healthcare team provided daily supervision [23,27], or used telephone calls to establish empathetic and personalized communication with patients and caregivers [13]. The role of the healthcare professional is essential in training patients to use the technology at home and in adjusting pain therapy, as highlighted by Glauser et al. [17] and Cheng et al. [4].

- Clinical Outcomes

In almost all of the reviewed studies, pain was assessed using validated and standardized scales (e.g., VAS, NRS, etc.), and in most cases a significant reduction in perceived pain was observed, along with a notable decrease in the use of opioid medications [4,13,28], and a consistent improvement in patient satisfaction [29]. Other outcomes also emerged from the studies:

- Improved sleep quality [22];
- Perceived quality and speed of post-surgery recovery [19,27];
- Reduction in emergency department visits [18];
- Greater patient autonomy in managing the postoperative period, as demonstrated by adherence to ERAS pathways [15,23];
- Faster acquisition of clinical data to support patients in making therapeutic decisions [7,14].

- Emerging Barriers

The use of technology naturally brings with it, especially among older patients, a number of barriers and limitations. The main issues reported include:

- Low digital literacy and difficulty in adopting modern technological aids among the elderly population [6,8];
- Unstable internet connections, lack of connectivity in certain geographic areas, or limited availability of devices [4,17];
- Discontinuity in adherence to the use of technology [5];
- In models based on less advanced technology, such as standard phone calls, loss to follow-up was observed [7].

Finally, many studies highlight the need for further randomized controlled trials (RCTs), with broader geographic distribution and more diverse populations. This is essential to confirm the effectiveness of available technologies and digital models, as well as to validate the sustainability of home-based pain monitoring [29,30].

## 4. Discussion

This study highlighted how the implementation of technology, digital tools, and telemedicine for the home-based management of postoperative pain is associated with symptom reduction, lower opioid consumption, and increased patient satisfaction. Specifically, among the 28 studies included in this review, 18 reported a reduction in postoperative pain, 13 highlighted an increase in patient satisfaction, and 4 described a decrease in opioid consumption. Several studies reported more than one outcome. These findings confirm the potential of digital interventions to improve both clinical and experiential outcomes in the postoperative period. The active role of healthcare professionals, particularly nurses, proved to be important for remote monitoring and patient support/education. The technologies used include mobile apps, remote messaging platforms, digital tools such as wireless sensors, and web-based systems. While the studies by Hofstad et al. [8] and Carlier et al. [3] reported a clear reduction in postoperative pain and improved functional recovery associated with the use of digital tools, Nilsson et al. [19] instead highlighted that some patients experience difficulties and psychological distress, suggesting that technology alone may not be sufficient. Similarly, McGillion et al. [24] reported a decrease in hospital readmissions, better pain control, and improved therapeutic appropriateness through remote monitoring and daily video calls with nurses. These contrasting findings suggest that although digital interventions can improve outcomes for many patients, their effectiveness may vary depending on individual factors such as emotional status, age, and digital literacy.

The active involvement of healthcare professionals appears crucial to ensure the successful integration of digital tools into healthcare systems, as also shown by Cheng et al. [4], who used a WeChat-based system to maintain constant contact with patients at home. Kane et al. [29] confirmed that telemedicine follow-ups are associated with high overall patient satisfaction and reduced resource use and waste.

Studies such as Nilsson et al. [25] focused on patient-reported experiences, showing that symptoms like fatigue or psychological discomfort can hinder home recovery even when technological support tools are available. Similarly, the study by Morgan et al. [18] emphasized the effective management of pain in elderly and vulnerable patients through mobile apps, also highlighting the value of these tools in providing health professionals with meaningful clinical data.

However, despite the positive aspects, several barriers emerged, including limited digital literacy [7], unstable internet access in certain geographical areas [9], and low adherence to digital programs among older and more vulnerable populations [22,27]. The literature also highlights several barriers to implementation. Cleeland et al. [33] noted that older patients with limited digital literacy often prefer telephone-based support over innovative web platforms, due to difficulties in using digital technologies. Similarly, McGillion et al. [23] reported low adherence to health apps among some users, emphasizing the need to strengthen initial education for both patients and caregivers. Azzellino et al. [34] proposed, in this regard, that structured discharge planning, combined with caregiver education and early activation of home care services, could support the adoption of digital tools and reduce hospital readmissions, particularly among frail populations. These findings suggest that future healthcare policymaking should include the development of more accessible tools and adequate training pathways, with the goal of improving digital health equity. Overall, the results indicate that, although digitalization offers significant potential for improving postoperative pain management—thanks to timely monitoring and reduced hospital readmissions—its actual impact strongly depends on the healthcare context, cultural background of the population, and continuity of care. Clinically, these tools appear to support pain relief and functional recovery; organizationally, they may reduce workload and resource utilization. However, variability in adherence, access to technology, and patients’ emotional responses limits the consistency of outcomes. Therefore, each implementation should be personalized, inclusive, and accompanied by adequate training, continuous evaluation, and political-institutional support.

In light of these critical issues, there is a need for innovative and integrated care models. Azzellino et al. [10] suggest that effectiveness does not come from a single action but from the synergy of coordinated interventions and the integration of clinical, relational, and social components. The integration of digital tools and telemedicine into home-based and post-surgical follow-up care appears to be a promising strategy to improve care quality, promote patient autonomy, and reduce healthcare costs. However, the success of these innovations in everyday clinical practice will depend on their ability to adapt to the care needs of frail patients, their digital literacy levels, and the training of healthcare professionals.

### 4.1. Implications for Clinical Practice

The review highlights several implications for clinical practice. The implementation in daily professional practice of digital tools for monitoring postoperative pain at home is emerging as a strategy to enhance continuity of care and improve patient assistance. The possibility of collecting clinical data and patient-reported symptoms in real time ensures an appropriate and timely clinical response, with high percentages of complication reduction and better pain control. Added to this is a potential reduction in opioid medications.

The use of technology, mobile applications, and automated systems allows for more flexible, sustainable, and personalized care management. However, the implementation of these technologies requires a careful assessment of digital literacy levels within healthcare organizations and therefore investments in digital training and technological infrastructure.

### 4.2. Limitations of the Study

This scoping review has some limitations. The included studies show a limited presence of randomized controlled trials, which restricts the ability to formulate strong clinical recommendations. Several studies had small sample sizes and an insufficient evaluation of psychological or organizational outcomes associated with the use of technology in the postoperative period. This highlights the need for future research and updates based on robust methodologies, capable of assessing the clinical, experiential, social, and economic effects of technology within care processes in an integrated manner.

## 5. Conclusions

This study confirms that the implementation of digital tools and telemedicine in the daily clinical practice for home-based pain management in postoperative patients represents a promising strategy to enhance hospital-to-community integration and continuity of care. The active involvement of healthcare professionals, particularly nurses, is identified as fundamental. The reviewed studies confirm improved pain control and a reduction in opioid use. However, barriers persist due to low digital literacy among frail and elderly patients, infrastructural limitations, and the need for proper staff training. These issues must be urgently addressed to ensure equitable access to care. Future studies should adopt more robust methodologies and include larger and more diverse populations, for example, studies with over 500 participants from both urban and rural settings and covering at least three different surgical specialties, to better assess the clinical, economic, and organizational impact of these digital tools. It should be noted, however, that the overall certainty of the evidence remains limited due to the diversity of study designs and the variability of clinical settings and populations. This variability limits the generalizability of the findings and highlights the need for more rigorous research.

## Figures and Tables

**Table 1:** Characteristics of the included studies (n = 28). The table reports author and year, country, study type, main topic, study population, key findings, and identified research gaps for each included study.

Author/ Year	Country	Study Type	Main Theme	Population	Key Findings	Research Gaps
Debono et al., 2016 [5]	France	Prospective observational study	Home monitoring with mobile app after outpatient lumbar discectomy, with automatic alert based on pain and other symptoms.	60 patients undergoing outpatient lumbar discectomy	31.7% activated alarms, mainly for postoperative pain (72.4%). The app allowed timely contact with clinicians, optimized pain management and avoided improper access. High patient satisfaction and good usability	Pilot study design; need for RCTs to compare efficacy versus traditional follow-up.
Wildemeersch et al., 2018 [15]	Belgium	Prospective cohort study with retrospective comparison	Creating an Enhanced Recovery Pathway (ERP) with eHealth technologies for postoperative pain monitoring.	29 adolescents underwent minimally invasive thoracic surgery for pectus	The use of a digital platform with telemonitoring devices has allowed for good long-term and home-based pain control. In addition, the platform allowed for early identification of persistent pain. High satisfaction and good adherence.	Small sample size; low adherence; single-center study; need for large-scale RCTs
Anthony et al., 2018 [16]	U.S.A.	Prospective study	Using an automated mobile phone messaging robot for postoperative patient monitoring	47 patients undergoing orthopaedic surgery (monitored for 7 days)	The use of the messaging robot has improved postoperative monitoring, facilitating communication between patients and healthcare professionals.	Monocentric study Small sample To be tested in larger populations and in different settings
Dahlberg et al., 2019 [1]	Sweden	Secondary analysis of multicenter RCT	Personalized postoperative follow-up via app (RAPP) with call activation by the patient.	494 adult patients undergoing outpatient surgery (intervention group).	17% of patients-initiated contact via the app, mainly for pain. Patients who requested contact had worse postoperative recovery. The app allows effective, personalized follow-up.	Limited data, missing demographic and clinical variables
Glauser et al., 2019 [17]	U.S.A.	Prospective pilot study	Introduction of NeuroPath mobile app to monitor postoperative phase of patients undergoing spontaneous surgery, with ERAS principles.	30 patients undergoing elective spine surgery.	Discharged patients used the app regularly and showed improvements in pain reduction, increased physical activity, and a tendency to discontinue opioids. The app allows real-time therapy monitoring, with active patient involvement.	Small sample size Low adherence to digital tool. Need for user training on interface Need for large-scale studies on clinical outcomes
Pickens et al., 2019 [18]	U.S.A.	Prospective pilot study	Use of a mobile application for the real-time collection of Patient-Reported Outcomes (PRO) in hepatopancreatobiliary surgery within an ERAS^®^ pathway	122 patients monitored for 30 days post-discharge	App adoption was 93% (114/122). Patients completed 62% of the PROs on quality of life, postoperative pain, nausea, opioid use, and adherence to ERAS^®^ pathway elements. During post-discharge follow-up, 12 patients reported that the app prevented a hospital call and 3 reported avoiding an emergency room visit.	Pilot study with limited generalizability; single-center study; need for larger confirmatory trials
Nilsson et al., 2019 [19]	Sweden	Mixed methods study (RCT + interviews)	Comparison between general anesthesia (GA) and regional anesthesia (RA) on the quality of postoperative recovery, assessed by mobile app (RAPP) and SwQoR questionnaire	401 patient’s outpatient surgery (quantitative), 20 patients (qualitative)	Pain from the surgical wound was the most frequent. The mobile app allows daily follow-up at home for 14 days. The quality of recovery was worse in patients with GA.	Swedish sample only. It turns out that not all patients used the technology.
Highland et al., 2019 [20]	U.S.A.	Pilot Randomized Controlled Trial	Feasibility of app-based monitoring of postoperative pain and anesthesia effects	50 adult patients	The mCare app was feasible for postoperative assessment. Nurses reported higher satisfaction with the app compared to standard telephone follow-up methods.	Need for larger studies, long-term efficacy, and broader applicability
Kane et al., 2020 [21]	U.S.A.	Prospective RCT	Postoperative follow-up via telemedicine after arthroscopic rotator cuff repair.	58 patients (28 with telemedicine follow-up, 30 with face-to-face follow -up)	No difference in pain scores between the groups at 2, 6 and 12 weeks. Patients in the telehealth group showed greater preference for this model, with less time spent and fewer working hours lost. Pain was monitored with VAS at each visit.	Monocentric design, short follow-up, limited generalizability due to inclusion of only tech-accessible patients.
Loring et al., 2021 [22]	U.S.A.	Prospective cohort pilot study	Using a non-contact wireless radio sensor (“Emerald”) to collect objective data on sleep quality and correlation with postoperative pain after laparoscopy for endometriosis.	Patients over the age of 18 with regular and independent sleep patterns and who are scheduled for laparoscopy for the diagnosis and treatment of suspected endometriosis.	The sensor collected objective physiological data for 10 weeks. There was a positive correlation between time to deep sleep and self-reported pain the following day. The device allowed for noninvasive and continuous monitoring of recovery.	Very limited sample size. Need for larger cohorts
Carlier et al., 2021 [3]	France	Monocentric retrospective observational study	Postoperative pain monitoring after ambulatory surgery via mobile app.	1691 adult and pediatric patients.	The app enabled personalized follow-up, early symptom detection, and targeted clinical interventions. High patient adherence and satisfaction.	Questionnaires not validated. Sample limited to 5 specialties. Lack of data on the functional and social impact of pain
McGillion et al., 2021 [23]	Canada	Multicenter RCT	Integrated post-discharge remote monitoring with telemedicine to reduce complications and manage symptoms, including pain.	905 adults ≥40 years discharged after non-elective surgery	The remote monitoring group reported less pain on days 7, 15, and 30. The system detected and corrected more errors in analgesic management. The intervention was well accepted and improved clinical follow-up at home.	Pain was not the primary outcome.
Cheng et al., 2021 [24]	China	Single-centre prospective observational study	Remote monitoring via app (WeChat) of post-operative symptoms after lung cancer surgery	826 patients undergoing lung surgery; 589 with at least 3 answers to questionnaires	The app allowed the monitoring of pain up to 12 weeks. Pain decreased from 4.1 to 2.2. Factors associated with increased pain: female sex, age >60, thoracotomy, operating time >90 min, drainage >7 days. High adherence to the programme, and effective data collection without staff overload	Exclusion of patients without smartphone or family support.
Tiozzo et al., 2021 [25]	Italy	Prospective comparative study	Using a mobile application to monitor postoperative pain at home in pediatric patients	487 pediatric patients	Forty-four percent of participants reported pain in the first 24 h after surgery, while 22% reported pain between the first and fifth days. The use of the app facilitated pain assessment, allowing healthcare professionals to actively involve pediatric patients and their parents in pain management at home.	Need for long-term evaluation of app effectiveness
Walrave et al., 2022 [26]	France	Multicenter prospective descriptive study	Use of a smartphone application for monitoring postoperative pain in children undergoing ambulatory surgery	Children undergoing outpatient surgery	The application has provided the opportunity to effectively monitor postoperative pain at home, improving communication between patients and healthcare professionals.	Need for long-term evaluation, limited generalizability
Rian et al., 2022 [6]	Norway	Usability and feasibility sub-study within RCT	Web-based tools (Eir) for pain assessment and home follow-up.	Patients undergoing total knee replacement (aged 32–78 years)	Most patients found the system easy to use and intuitive. The interface was accessible even to patients with limited technological skills. Most participants used the system without requiring technical support. The difficulties encountered were mainly related to the internet connection or login. The tool allowed for valid clinical follow-up with effective, constant, and precise recording of symptoms and use of analgesics, providing useful data for clinical practice and research. The system has proven itself in home monitoring of patients after hospital discharge. Older patients and those who did not have an electronic device had greater difficulty. Therefore, technological experience had a greater impact than the complexity of the tool itself.	Small sample size; feasibility sub-study; low digital literacy; barriers in older adults; infrastructure limitations.
Wood et al., 2022 [14]	Canada	Qualitative study	Identification of risk factors for postoperative pain and selection of digital tools for monitoring and data collection.	22 participants (clinicians and caregivers of operated children)	Five key domains were identified: demographics, psychosocial, clinical, PREMs, and PROMs. The study also defined functional requirements and delivery modalities (e.g., electronic and repeated instruments). A data collection system was designed for future personalized predictive models.	Small sample size; lack of quantitative data.
Tran-McCaslin et al., 2022 [13]	U.S.A.	Retrospective observational study on pilot program	Same-day discharge (SDD) program with remote monitoring via daily phone calls for postoperative pain control and opioid use after minimally invasive colorectal surgery	37 patients selected for good health and elective colorectal surgery	Patients used less opioids than expected, despite reporting moderate pain in the first few days. The intervention led to a reduction in opioid prescriptions (from 40 to 10 tablets). Daily telephone calls allowed continuous clinical monitoring, including pain assessment and instructions on multimodal therapy.	Small, highly selected sample Limited to telephone-based monitoring Need for further studies to support generalizability
Hansen et al., 2022 [9]	Denmark	Retrospective comparative observational study	Reducing patient-operator contact in the fast-track after total hip replacement and impact on patient-reported outcomes, including pain	Patients undergoing THA in three different fast-track pathways (with various levels of clinical contact, including telephone follow-ups)	The reduction of patient-staff contact (e.g., replacement by phone calls) did not affect self-reported postoperative pain or mobility. Satisfaction remained high.	Single-centre study
Gille et al., 2022 [7]	Belgium	Narrative revision of literature	Postoperative follow-up systems after peripheral nerve blocks in ambulatory surgery, with a focus on digital instruments	67 articles included, selected from international databases (Scopus, Embase, MEDLINE)	Patients receiving peripheral nerve blocks may experience moderate to severe pain up to 78% on the first postoperative day. Telephone follow- up has high rates of loss to follow-up (up to 50%). Digital systems (automated SMS, apps, video visits) improve response rates and detection of adverse events, including pain and neurological complications.	Few studies assess the impact on outcomes such as pain or readmissions.
McLemore et al., 2022 [2]	U.S.A., Canada, France	Multi-centre narrative review with clinical experiences	Digital follow-up and same-day discharge after minimally invasive colorectal surgery with pain monitoring	Patients undergoing minimally invasive colorectal surgery (multi-centre experience)	Pain was managed and monitored at home using NRS scales. Digital technologies (apps, video calls, phone calls) enabled active and timely follow-up. Reduced opioid use and high patient satisfaction were observed.	Need to validate effectiveness and sustainability of the model in larger populations.
Thiel et al., 2023 [27]	The Netherlands	Unblinded multi-centre RCT protocol	Remote monitoring of postoperative recovery via app after day surgery	310 adult patients undergoing outpatient surgery in three Dutch hospitals	The app allows daily recording of pain and nausea; in case of need, the patient is contacted by healthcare professionals trained in empathic communication. An improvement in the quality of perceived recovery, measured by QoR-15 on day 7, is assumed.	Study still ongoing. No definitive clinical results available. Excluding elderly patients or those with low digital literacy.
Morgan et al., 2023 [28]	U.S.A.	Mixed Methods Feasibility and Acceptability Pilot Study	Use of a mobile app (CPMRx) for postoperative pain management and monitoring of opioid use at home	10 elderly patients with an average age of 68 8 years	The mobile app was easily used by participants and was considered useful for postoperative pain management. The commitment to using the app, despite the age, was high. No problems emerged in using the technology. It emerged that the app could help personalize and optimize pain treatment, especially with the consumption of opioid drugs. The app provides tools for therapeutic education and supports patients in the decision before taking drugs, promoting a more conscious and safe use. The app collects data and therefore can offer clinically relevant information to improve pain management and therapy, preventing risks related to the use of opioids.	Small sample, low diversity, need for broader settings
Weiss et al., 2024 [29]	U.S.A	Prospective observational study	Using a mobile digital health tool for monitoring and support during post-operative recovery after radical cystectomy.	Adult patients undergoing radical cystectomy	The app was found to be accessible and easy to use, well accepted by patients, and useful for daily symptom monitoring, including postoperative pain. It facilitated communication, educational support, and symptom management at home	Small sample size; lack of RCTs; unclear impact on clinical outcomes and long-term pain
Hofstad et al., 2024 [8]	Norway	Prospective cohort study	Home follow-up via a web-based digital tool (EIR) to monitor pain and quality of life in the 30 days after total hip arthroplasty via a web-based digital tool (EIR)	82 adult patients undergoing elective primary THA	Pain and quality of life remain critical in the first week after discharge but progressively improve. After 30 days, 32% of patients are still using opioids. The use of the web-based tool has allowed continuous monitoring, with high adherence.	Single-center study; exclusion of patients with poor digital skills or no home connection
Levit et al., 2024 [30]	Not-available	Systematic review of RCTs	Effectiveness of virtual (VR) and augmented reality (AR) in surgical pain management	Patients undergoing various surgeries	VR/AR have shown a significant reduction in postoperative pain in several studies, with decreased analgesic use and increased patient satisfaction.	Need for RCTs with larger samples and VR/AR protocol standardization
Vitale et al. (2025) [31]	Spain	Single-centre prospective observational study	Mobile app for monitoring acute postoperative pain in major outpatient surgery	Patients undergoing major outpatient surgery (exact number not reported in abstract)	The app was found to be feasible and acceptable. It allowed real-time monitoring of acute postoperative pain. High prevalence of moderate-to-severe pain and low satisfaction with analgesic treatment were observed, suggesting suboptimal pain control and the need for better guideline adherence.	Single centre; no control group; low satisfaction with analgesia despite technological support
Brintz et al. (2025) [32]	USA	Prospective intervention study	Telehealth-delivered mindfulness-based intervention post-lumbar spine surgery	Patients recovering from lumbar spine surgery (sample size not specified in summary)	The intervention was feasible and well accepted. Participants showed clinically meaningful improvements in pain intensity and disability at 3 months. Modifications to content and delivery were made based on patient feedback.	No control group; results may not be generalizable beyond lumbar spine surgery; sample size not reported

## Data Availability

No new data were created or analyzed in this study.

## Supplementary Materials

The following supporting information can be downloaded at: https://www.mdpi.com/article/10.3390/jcm14114009/s1, Supplementary File S1: Search strategy.
