# Supplementary material for: Use of Digital and Telemedicine Tools for Postoperative Pain Management at Home: A Scoping Review of Health Professionals’ Roles and Clinical Outcomes
Source: J Clin Med. 2025 Jun 5;14(11):4009. doi: 10.3390/jcm14114009 (PMC12156900; doi:10.3390/jcm14114009)
Supplement: Supplementary file 1 [file jcm-14-04009-s001.zip › jcm-3609196-supplementary.pdf]

Supplementary File S1 Search strategy

PUBMED

("postoperative pain" OR "surgical pain") AND ("telemedicine" OR "telehealth" OR "digital health" OR "eHealth" OR "mHealth" OR "mobile app*" OR "remote monitoring" OR "virtual care" OR "wearable device*") AND ("home care" OR "home setting" OR "post-discharge" OR "outpatient care" OR "ambulatory care") AND ("health professional*" OR "nurse*" OR "physician*" OR "clinician*" OR "healthcare provider*") AND ("child*" OR "adolescent*" OR "adult*" OR "pediatric*" OR "all ages")

("postoperative pain" OR "surgical pain") AND ("telemedicine" OR "digital health" OR "telemonitoring" OR "mobile app*" OR "eHealth" OR "virtual care") AND ("home" OR "after discharge" OR "home-based care" OR "outpatient") AND ("healthcare provider*" OR "medical team" OR "nurse*" OR "physician*") AND ("child*" OR "adolescent*" OR "adult*" OR "older adult")

("postoperative pain" OR "surgical pain") AND ("telemedicine" OR "telehealth" OR "digital health" OR "eHealth" OR "mHealth" OR "mobile application*" OR "remote monitoring" OR "virtual care" OR "wearable device*") AND ("home" OR "post-discharge" OR "after discharge" OR "home-based" OR "ambulatory")

SCOPUS

TITLE-ABS-KEY ("postoperative pain" OR "surgical pain") AND TITLE-ABS-KEY ("telemedicine" OR "telehealth" OR "digital health" OR "eHealth" OR "mHealth" OR "remote monitoring" OR "mobile app*" OR "virtual care" OR "wearable device*") AND TITLE-ABS-KEY ("home care" OR "post-discharge" OR "home setting" OR "outpatient care" OR "ambulatory care") AND TITLE-ABS-KEY ("health professional*" OR "nurse*" OR "physician*" OR "clinician*" OR "healthcare provider*") AND TITLE-ABS-KEY ("child*" OR "adolescent*" OR "pediatric*" OR "adult*" OR "elderly" OR "all ages")

TITLE-ABS-KEY ("postoperative pain") AND TITLE-ABS-KEY ("telemedicine" OR "digital health" OR "remote monitoring" OR "virtual care" OR "mobile app*" OR "eHealth") AND TITLE-ABS-KEY ("home" OR "home-based care" OR "after discharge" OR "outpatient") AND TITLE-ABS-KEY ("child*" OR "adolescent*" OR "pediatric*" OR "adult*" OR "older adult*")

WEB OF SCIENCE

TS=("postoperative pain" OR "surgical pain") AND TS=("telemedicine" OR "telehealth" OR "digital health" OR "eHealth" OR "mHealth" OR "remote monitoring" OR "mobile app*" OR "virtual care" OR "wearable device*") AND TS=("home care" OR "post-discharge" OR "home setting" OR "outpatient care" OR "ambulatory care") AND TS=("health professional*" OR "nurse*" OR "physician*" OR "clinician*" OR "healthcare provider*") AND TS=("child*" OR "adolescent*" OR "pediatric*" OR "adult*" OR "elderly" OR "all ages")

TS=("postoperative pain") AND TS=("telemedicine" OR "digital health" OR "eHealth" OR "remote care" OR "virtual care") AND TS=("home" OR "home setting" OR "post-discharge" OR "outpatient") AND TS=("child*" OR "adolescent*" OR "pediatric*" OR "adult*" OR "older adult")
